# First observations on the life cycle and mass eclosion events in a mantis fly (Family Mantispidae) in the subfamily Drepanicinae

**DOI:** 10.3897/BDJ.5.e21206

**Published:** 2017-11-22

**Authors:** James B Dorey, David J Merritt

**Affiliations:** 1 Flinders University, Adelaide, Australia; 2 The University of Queensland, Brisbane, Australia

**Keywords:** mantis flies, campodeiform larvae, Mantispidae, Drepanicinae

## Abstract

**Background:**

The Mantispidae are a distinctive group of Neuroptera known for the adults’ possession of raptorial forelegs. There are four recognised, extant subfamilies of Mantispidae: the Mantispinae, Symphrasinae, Calomantispinae and Drepanicinae. The life history and larval behaviour of the subfamily Mantispinae is best known: the immatures are spider egg predators. Among the three remaining subfamilies, larval Symphrasinae and Calomantispinae most likely predate on other small arthropods, while the immature life history of Drepanicinae, until now, remained completely unknown.

**New information:**

Here we provide observations of annual, near-synchronised, mass emergences of adults of the drepanicine, *Ditaxis biseriata* (Westwood), within a well-established *Macadamia* orchard in northern New South Wales, Australia. A female deposited fertile eggs, allowing this first report of egg batch and first instar morphology. The mass emergence of mobile pharate adults from the ground was observed in the same month in two consecutive years. The pharates climbed tree-trunks for a distance before undergoing eclosion. The newly-hatched first instar larvae are campodeiform and prognathous; a typical morphology among Mantispidae. After hatching, they drop to the ground and burrow into soil. They are unpigmented and appear to lack stemmata. Together, the observations infer that the immature component of the life cycle takes place underground in forested habitats. If this feature is common among the Drepanicinae, it might explain why so little is known of the biology of the immature stages.

## Introduction

The Neuroptera is one of the most ancient orders of insects that show complete metamorphosis. Larvae are typically predaceous with elongate, slender mouthparts that are adapted for piercing and sucking (Tauber et al. 2003). Within Neuroptera, the Mantispidae derives its name from its morphological resemblance to Mantodea (praying mantids), based on the raptorial forelimbs and elongated prothorax (Jepson et al. 2013, Liu et al. 2015). This resemblance is a case of convergent morphology evolved to suit the predatory behaviour exhibited by both groups (Redborg 1998). The family Mantispidae is composed of four extant subfamilies; Mantispinae, Symphrasinae, Calomantispinae and Drepanicinae (Liu et al. 2015, Lambkin 1986). Mantispinae is the subfamily whose biological traits are best-known (Redborg 1998, Ohl 2004); the immatures are exclusively spider egg predators during their development. The first instar larva is campodeiform and agile to enable host-finding while subsequent larval stages are scarabeiform. Larvae locate and attach to a spider and enter the spider’s egg sac either upon its construction or afterwards. Once inside, the larvae pierce and drain the spider eggs, undergoing three larval stages within the sac (Redborg and MacLeod 1984).

Compared to Mantispinae, knowledge of the morphology, biology and ecology of immatures of the other subfamilies of Mantispidae is “sketchy and fragmentary” (Redborg 1998) and that of Drepanicinae—the subject of this report—was heretofore completely unknown. From the limited information available, the feeding habits of larval Symphrasinae and Calomantispinae larvae appear to be fairly broad compared to the spider egg association of Mantispinae. Larvae of *Plega* spp. (Symphrasinae) have been reared in the laboratory on immature Lepidoptera, Hymenoptera and Coleoptera (Redborg and MacLeod 1984). In some species, the larvae predate upon larvae of bees or wasps, either social or solitary (Dejean and Canard 1990, Maia-Silva et al. 2013, Parker and Stange 2012). Larvae of Calomantispinae appear to predate on other arthropods. *Nolima pinal* larvae have been reared to adulthood on prey items that included larval Diptera, Hymenoptera, Lepidoptera and Coleoptera in addition to spider eggs and paralyzed spiders that had been removed from sphecid cells (MacLeod and Redborg 1982). Thus, it appears that, within Mantispidae, larvae of Mantispinae are specialist spider egg predators; whereas, larval Symphrasinae and Calomantispinae range from generalist to specialist arthropod predators.

This report provides information on the motility of pharate adults and adult eclosion behaviour of the Australian drepanicine mantispid, *Ditaxis biseriata* and the morphology of first instar larvae that emerged from a batch of deposited eggs is described. The observations were made possible by mass adult eclosion events occurring in a *Macadamia* orchard in northern New South Wales, Australia, at the same time of year over two consecutive years.

## Materials and methods

### Location

The study site was a *Macadamia* orchard, planted in 1988, located near Newrybar, NSW, Australia. It is located in a region of New South Wales colloquially known as “the Big Scrub”. Prior to European settlement, the region encompassed the largest area of subtropical rainforest in Australia. In the 19^th^ century, extensive timber-getting and clearing for agriculture took place; consequently, remnant rainforest is patchy and a number of rainforest regeneration projects are underway. The region is recognised as a biodiversity hotspot by the Australian Government Department of Environment and Energy. Parts of the orchard have a closed canopy and moist soil with abundant moss and other epiphytes on the macadamia trees. The northwest corner of the orchard has the most complete canopy, most epiphytes and dampest soil. The orchard where *D. biseriata* were observed was sprayed with pesticide (dipterex 500 sl) between 11^th^ and 16^th^ September 2015. This appeared to have no effect on subsequent emergences of pharate adults.

Observations of adult eclosion were made between 5^th^ to 22^nd^ September 2015 and from 17^th^ to 24^th^ September 2016. Mature adults and pharate adults were collected on and after 5^th^ September 2015.

### Methods

Some adults were kept in captivity (in petri dishes or insect containers) while others were killed and pinned. Pharate adults were preserved in ethanol.

Both field and studio photographs were taken, with studio images being of both live and preserved specimens. To record larval morphology, ethanol-preserved first instars were washed in water and cleared in KOH before slide-mounting and photomicroscopy.

For the timelapse observations of adult eclosion, a Canon EOS 60D dSLR camera with 100 mm macro lens was mounted on a tripod and focused on a pharate adult and 759 images were taken over a period of 43 minutes. The camera was controlled by an external remote control and a canon speedlite 580EX II was used to light the specimen. A video was produced comprising of all 759 images played at 25 frames per second.

## Taxon treatments

### Ditaxis biseriata

(Westwood, 1852)

#### Materials

- **Type status:** Other material. **Occurrence:** catalogNumber: JD1DB; recordedBy: James B Dorey; individualCount: 1; lifeStage: adult; associatedMedia: http://www.jamesdoreyphotography.com.au/Nonpublic-galleries/Mantispids/n-s3NpJH/; **Taxon:** taxonID: urn:lsid:biosci.ohio-state.edu:osuc_names:275502; scientificName: Ditaxis biseriata; kingdom: Animalia; phylum: Arthropoda; class: Insecta; order: Neuroptera; family: Mantispidae; genus: Ditaxis; **Location:** country: Australia; stateProvince: New South Wales; locality: Newrybar; verbatimElevation: 11 m; locationRemarks: label transliteration: "Newrybar, NSW, 28˚43'52.0"S 153˚33'18.8"E, J.B.Dorey, 05/09/2015, JD1DB"; verbatimCoordinates: 28˚43'52.0"S 153˚33'18.8"E; decimalLatitude: -28.731111; decimalLongitude: 153.555222; georeferenceProtocol: label; **Identification:** identifiedBy: Kevin Lambkin; dateIdentified: 2017; **Event:** samplingProtocol: sweeping; **Record Level:** language: en; collectionID: JD1DB; collectionCode: Insects; basisOfRecord: PreservedSpecimen
- **Type status:** Other material. **Occurrence:** catalogNumber: JD2DB; recordedBy: James B Dorey; individualCount: 1; lifeStage: adult; associatedMedia: http://www.jamesdoreyphotography.com.au/Nonpublic-galleries/Mantispids/n-s3NpJH/; **Taxon:** taxonID: urn:lsid:biosci.ohio-state.edu:osuc_names:275502; scientificName: Ditaxis biseriata; kingdom: Animalia; phylum: Arthropoda; class: Insecta; order: Neuroptera; family: Mantispidae; genus: Ditaxis; **Location:** country: Australia; stateProvince: New South Wales; locality: Newrybar; verbatimElevation: 11 m; locationRemarks: label transliteration: "Newrybar, NSW, 28˚43'52.0"S 153˚33'18.8"E, J.B.Dorey, 05/09/2015, JD2DB"; verbatimCoordinates: 28˚43'52.0"S 153˚33'18.8"E; decimalLatitude: -28.731111; decimalLongitude: 153.555222; georeferenceProtocol: label; **Identification:** identifiedBy: Kevin Lambkin; dateIdentified: 2017; **Event:** samplingProtocol: sweeping; **Record Level:** language: en; collectionID: JD2DB; collectionCode: Insects; basisOfRecord: PreservedSpecimen
- **Type status:** Other material. **Occurrence:** catalogNumber: JD3DB; recordedBy: James B Dorey; individualCount: 1; lifeStage: adult; associatedMedia: http://www.jamesdoreyphotography.com.au/Nonpublic-galleries/Mantispids/n-s3NpJH/; **Taxon:** taxonID: urn:lsid:biosci.ohio-state.edu:osuc_names:275502; scientificName: Ditaxis biseriata; kingdom: Animalia; phylum: Arthropoda; class: Insecta; order: Neuroptera; family: Mantispidae; genus: Ditaxis; **Location:** country: Australia; stateProvince: New South Wales; locality: Newrybar; verbatimElevation: 11 m; locationRemarks: label transliteration: "Newrybar, NSW, 28˚43'52.0"S 153˚33'18.8"E, J.B.Dorey, 05/09/2015, JD3DB"; decimalLatitude: -28.731111; decimalLongitude: 153.555222; georeferenceProtocol: label; **Identification:** identifiedBy: Kevin Lambkin; dateIdentified: 2017; **Event:** samplingProtocol: As pharat adult; **Record Level:** language: en; collectionID: JD3DB; collectionCode: Insects; basisOfRecord: PreservedSpecimen

#### Description

A description of the subject of this study, *Ditaxis biseriata*, is provided by Lambkin (1986).

#### Diagnosis

*Ditaxis biseriata* was distinguished from its sister species, *Ditaxis meridiei*, by characters of the adult vertex and colouration of the sclerites (Lambkin 1986).

#### Distribution

The observation and collection site used in this study is within the distribution area described by Lambkin (1986), which covers the east coast of Queensland and north-east coastal region of New South Wales, Australia.

#### Biology

Pharate adults and eclosion behavior

The location within the orchard with the highest density of *D. biseriata* is wetter and has a more complete canopy then the rest of the orchard and is close to a 30-year old regenerated rainforest patch. Evening observations in early September found between one and five *D. biseriata* eclosing on most trees, while later in September very few were found, indicating that the mass eclosion is probably restricted to a few weeks of the year. Upon searching, eclosing adults were found in the regenerated rainforest patch near the *Macadamia* orchard, but at much lower densities.

**Figure 1.**
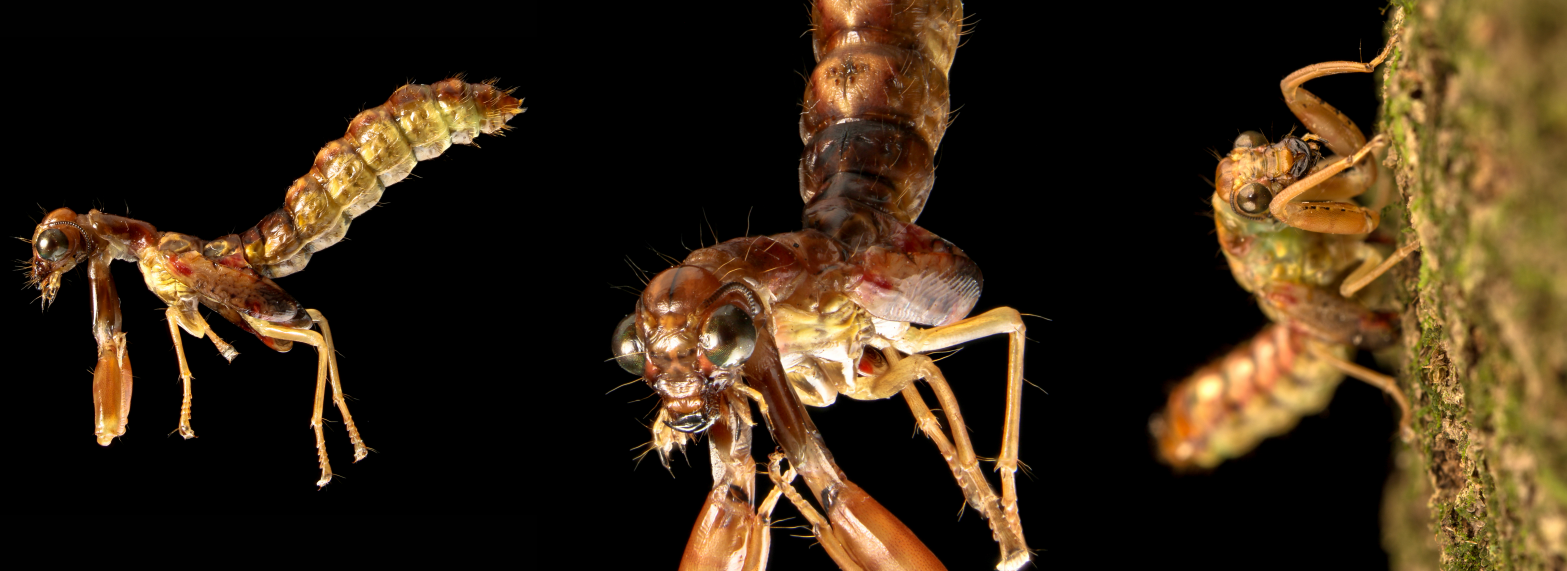
Pharate adult of *Ditaxis biseriata.* Left. Lateral view. Middle. Foreshortened frontal view. Right. A pharate adult at the immobile stage just prior to the initiation of adult eclosion, grasping a tree trunk.

Pharate adults (Fig. 1) were first observed walking upward on tree-trunks soon after sunset and would continue to appear for several hours, but in progressively lower numbers. Adult eclosion took place on tree trunks at heights that varied from ground level to approximately 2 m. Some individuals were observed to eclose on the ground or even on a camera tripod. The few individuals that eclosed on the ground appeared to be smaller than average. Pharate adults that were removed and placed on the ground would resume movement up a nearby tree to eclose. The adults are approximately 21 mm long, measured from head to tip of abdomen.

**Figure 2.**
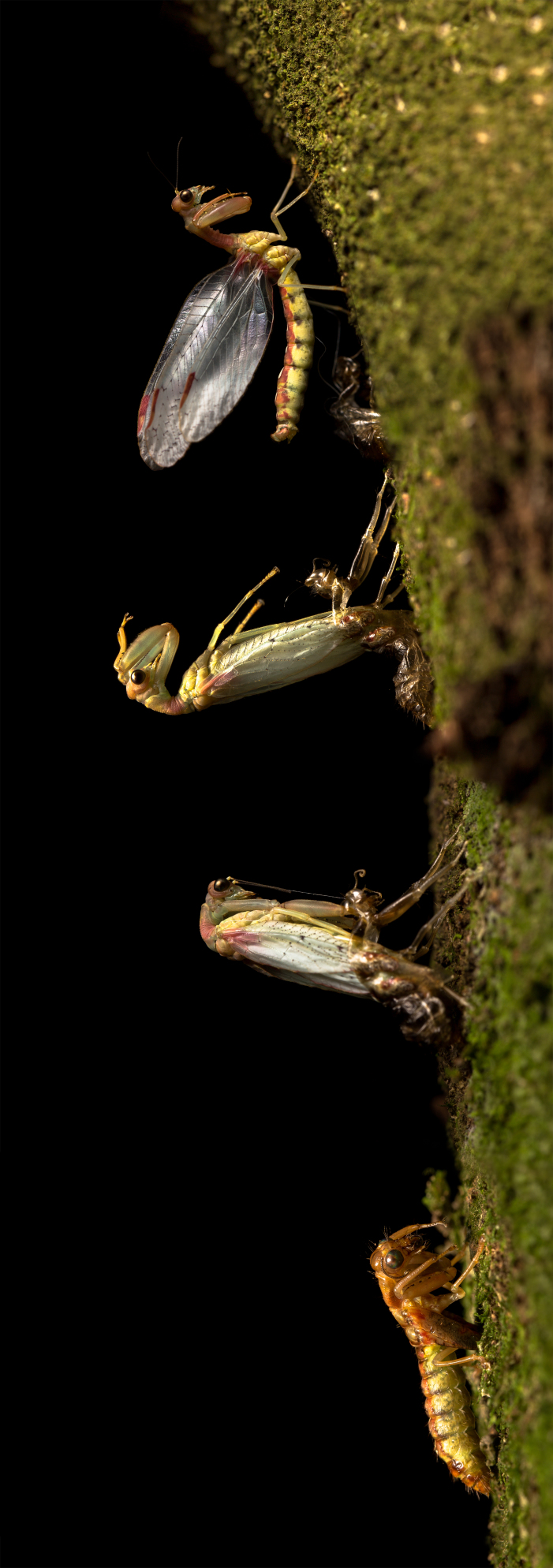
Eclosion of *Ditaxis biseriata*. The images, from bottom to top, show a pharate stage when it becomes stationary before eclosion begins (bottom), an adult in the process of extracting the wings and legs from the pupal case, an adult with its legs free of the pupal case and (top) an adult with its wings in the splayed condition progressively becoming transparent.

Four steps in the process of eclosion are depicted in Fig. 2 and a timelapse video of eclosion of a single adult is shown in Fig. 5. At eclosion, pharate adults stopped moving and began to pump and flex their abdomen. The pupal cuticle split along the thoracic midline and the adult’s thorax and head emerged with the head flexed ventrally. Once the head and legs were drawn free, the adult body rotated backwards over the pupal case, remaining attached to the pupal case at the posterior abdomen. Then the adult reached forward, grasped the tree trunk with all legs and moved ahead a few steps to completely free its body from the pupal case. The adult then released the grasp of the fore-legs and flexed backwards at the thorax-abdomen junction so that the head and thorax were held horizontally while the abdomen remained vertical, holding to the tree-trunk via the 2^nd^ and 3^rd^ pairs of legs. The prothoracic legs were retracted into the typical mantid-like position. The wings expanded and progressively became transparent in all but the pigmented sections. This wing clarification initiated proximally and proceeded to the distal wingtips. When it was almost complete, the adult straightened at the thorax-abdomen junction to adopt the typical adult posture and the wings transitioned from a splayed arrangement to the typical adult tent-like arrangement. If mechanically disturbed or exposed to flash photography before the abdominal peristalsis movements were initiated, pharates would not eclose. However, once peristaltic movements were initiated, they would complete eclosion. Predation of the newly-eclosed, immobile adults by spiders or ants was occasionally observed. Few adults were observed at the study site other than those that had just eclosed, possibly due to adults flying away from the site or into the canopy.

Eggs and first instar larvae

**Figure 3.**
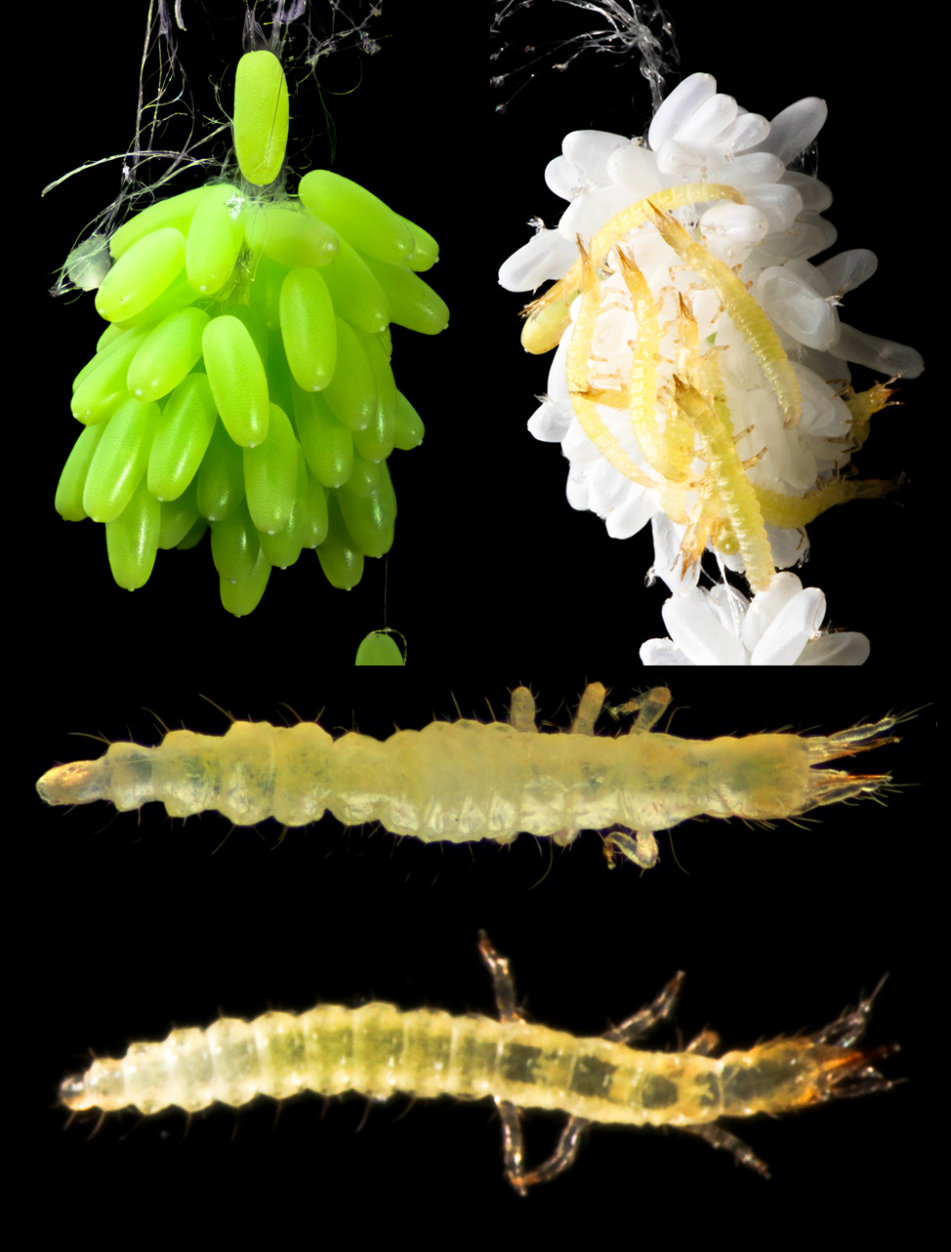
*Ditaxis biseriata* eggs and larvae. Top Left. Egg batch before eclosion; Top Right: egg cluster after eclosion of first instars, seen clustered on the egg batch; Middle: dorsal view of preserved first instar; Bottom: dorsal view of live first instar during locomotion.

An adult female collected on 5th September 2015 laid c. 120 eggs attached to the lid of a petri dish two days after collection. The lime-green eggs, each c. 0.83 mm long, were deposited in a cluster suspended on a cord of diffuse silken threads (Fig. 3). Upon hatching at 12 days after deposition, larvae clung to the egg cluster for a period (Fig. 3) before dropping to the bottom of the petri dish. They were then able to climb up and out of the petri dish and wander, dropping to the ground whenever they encountered a ledge. When larvae were introduced to loose soil they would burrow into it.

**Figure 4.**
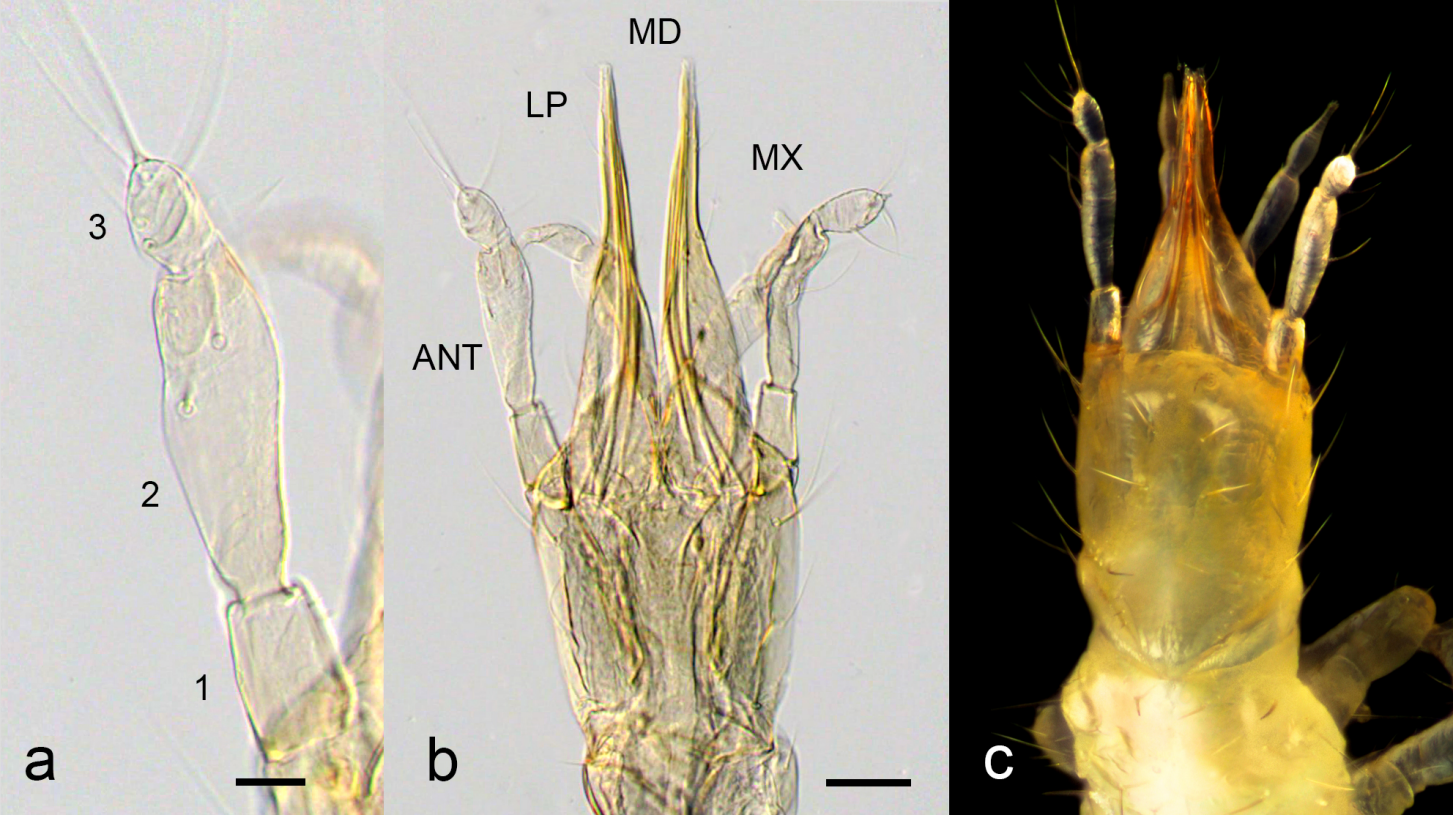
Larval head of *Ditaxis biseriata*. a and b. Cleared whole-mount of first instar larval antenna (a) and head (b). The antennal segments are numbered from proximal to distal. The head structures depicted in (b) include the antennae (ANT), labial palps (LP), mandibles (MD) and maxillae (MX). c. Dorsal view of the head of a preserved first instar larva. Scale bar in (a) is 20 microns and in (b) 50 microns.

The larvae are campodeiform, approximately 2 mm long, generally unsclerotised and creamy-white to light brown in colour. The mouthparts and parts of the head capsule are lightly sclerotized. The head capsule is longer than it is broad with distinctive prognathous mouthparts and anteriorly-directed antennae and labial palps (Fig. 4). The ipsilateral mandibles and maxillae combine to form paired piercing/sucking components projecting anteriorly. In life, they are generally held together along the midline.

The larval antennae are 3-segmented with a lobed terminal segment (Fig. 4). Multiple sensilla are present on the antennae including a pair of terminal sensilla extending from the tip of each distal antennomere. The terminal segment of each labial palp narrows distally to form a finger-like terminal projection. No stemmata were distinguishable in cleared specimens, and no pigmentation associated with stemmata could be seen in living specimens or in uncleared specimens stored in ethanol.

## Discussion

### Mass eclosion

As far as the authors are aware, mass, near-synchronous emergence of mantispids is a previously unreported phenomenon. It is uncertain if the emergence in such large numbers is a natural occurrence or an artefact of monoculture farming. Adults and exuviae were observed in other local *Macadamia* orchards at around the same time as the emergence near Newrybar (pers. comm. Jarah Coates and Ken Dorey) and emergence was observed simultaneously in nearby regenerated rainforest, suggesting that emergence is highly seasonal over the local area, perhaps through detection of photoperiod or some attribute of their yet-unknown food source, and that the life cycle covers a single year. Further, the presence in rainforest as well as in a location within the orchard with high soil moisture, dense canopy cover and numerous epiphytes hints at a rainforest association. The much higher prevalence of emergence in the *Macadamia* orchard compared to nearby regenerating rainforest could be due to more favourable conditions in the *Macadamia* orchard. It is possible that the coordinated emergence of *D.* biseriata facilitates mating because at least one of the females collected at the site produced fertile eggs. The species might not just be associated with rainforest; reports suggest that it is also common at the same time of year in open eucalyptus forest.

### Pharate adult behaviour

Pharate adults are remarkably mobile. They use negative geotaxis, possibly combined with visual orientation, to detect and move upward on a tree-trunk or other vertical object before becoming immobile and initiating eclosion behaviour. In Mantispidae, pupation generally takes place within a silken cocoon in a concealed location and the pharate adults leave the cocoon and walk some distance away before eclosing (Redborg 1998). In the Symphrasinae, pharate adults have been reported to emerge from the cocoon within nests of hymenopteran hosts from where they wander or are removed from the hive before they become immobile and eclose (Buys 2008, Maia-Silva et al. 2013). More widely, exarate pupae with mobile pharate stages are the norm in the clade Neuropterida (Kristensen 1999). In the raphidiopteran, *Raphidia bicolor*, motile pharate adults climb and face upwards on a vertical surface prior to eclosion (Kovarik et al. 1991). In the small, archaic family, Berothidae, sister family to the Mantispidae (Winterton et al. 2010), motile pharate adults were observed to break out of the cocoon and climb away to undergo the final ecdysis (Brushwein 1987). In the present case, cocoons were not observed but they would most likely be underground. The behaviours associated with adult emergence are typical of the Neuropterida with pharate adults tending to grasp a vertical substrate and adopt a posture that presumably allows the wings to expand and harden, as also seen in the Raphidophorid, *Raphidia bicolor* (Kovarik et al. 1991). The agility and behavioural repertoire of the pupa add substance to Kristensen's (Kristensen 1999) observations that the the motile, exarate, decticous pupae of related, basal endopterygotes—Aspoeck et al. (2012) called them "running pupae"—are substantially different to the compact, mostly inert pupal form of more derived endopterygotes.

### Eggs and larvae

The cluster of eggs suspended on silken threads is similar to the egg batch deposited by *Lomamyia lattipennis* (Berothidae) (Brushwein 1987, Toschi 1964). In one instance, larvae of *D. biseriata* were obtained by the same means: a captive female laying a batch of eggs that hatched (Winterton, personal communication). Larval morphology was not detailed but it was noted that they lacked stemmata (Winterton et al. 2010), an observation supported by this work. The newly hatched first instar larvae of *D. biseriata* are campodeiform and mobile with straight, anteriorly-directed mouthparts. Morphologically, they resemble the first instar larvae of other subfamilies of Mantispidae (Hoffman and Brushwein 1992) and the Berothidae (Hörnschemeyer 2013, Wedmann et al. 2013). Comparing the *D. biseriata* larva to larvae of Mantispinae (Hoffman and Brushwein 1992), the overall body shape and the head of *D. biseriata* are less robust and more elongate. Another difference is that *D. biseriata* has none of the pigmentation and sclerotisation usually seen in first instars of Mantispinae (Hoffman and Brushwein 1992, Redborg 1998, Redborg and MacLeod 1984). Distinctive features of the *D. biseriata* head include the elongate mouthparts and the lobulate rather than elongate terminal antennomeres and palpomeres. In head and mouthpart structure, as well as overall body shape, they resemble larval Berothidae (Wedmann et al. 2013), whose larvae appear to be specialist termite predators (Tauber and Tauber 1968). The antennae are not as slender and the terminal antennal sensillum is not as well developed as seen in fossil and extant Berothidae (Gurney 1947, Hörnschemeyer 2013, Toschi 1964).

Together, the behaviour and morphology of larvae, including their lack of stemmata, their tendency to drop from the egg and burrow into soil, and the subterranean origin of the pharate adults, support the premise that the larval and pupal stages of *D. biseriata* are subterranean. If this life history was common to all or most Drepanicinae, it could explain the lack of historical knowledge about the immature stages of the subfamily. The larval diet remains unknown; they could be subterranean arthropod predators like the subfamilies Symphrasinae and Calomantispinae or spider-egg predators like their sister sub-family, Mantispinae. A clue, at least in this species, might lie in the apparent preference for moist soil and the rainforest association. Future studies could focus on taking soil samples in the orchard to determine what potential prey or host species are present and possibly to detect larvae or pupae in situ.

## Supplementary Material

XML Treatment for Ditaxis biseriata

## Figures and Tables

**Figure 5.** Timelapse movie of an adult *Ditaxis biseriata* emerging from the pupal case. The pupa climbs a tree trunk and becomes still, then the adult emerges and its wings inflate and become transparent. In real time the process shown here took 43 minutes.
